# Long-term effects of Hirschsprung disease in adults: meta-analysis and patient-level regression study

**DOI:** 10.1093/bjsopen/zraf107

**Published:** 2025-11-04

**Authors:** Marta de Andres Crespo, Cornelia Byström, Athanasios Tyraskis, Annika Mutanen, Pernilla Stenström, Esther Hartman, Johan Danielsson, Simon Eaton, Paolo De Coppi, Anna Löf Granström, Tomas Wester, Mikko Pakarinen, Joe Curry, Stavros Loukogeorgakis, Joseph Davidson

**Affiliations:** Institute of Child Health, University College London, London, UK; Department of Pediatric Surgery, Karolinska Institutet, Stockholm, Sweden; Specialist Neonatal and Paediatric Surgery, Great Ormond Street Hospital, London, UK; Department of Pediatric Surgery, New Children’s Hospital, Helsinki, Finland; Department of Pediatric Surgery, University of Helsinki, Helsinki, Finland; Department of Pediatric Surgery, Lund University, Lund, Sweden; Department of Medical and Clinical Psychology, Tilburg University, Tilburg, the Netherlands; Institution of Women’s and Children’s Health, Uppsala University, Uppsala, Sweden; Institute of Child Health, University College London, London, UK; Department of Paediatric Surgery, Institute of Child Health, London, UK; Institute of Child Health, University College London, London, UK; Specialist Neonatal and Paediatric Surgery, Great Ormond Street Hospital, London, UK; Department of Paediatric Surgery, Institute of Child Health, London, UK; Department of Pediatric Surgery, Karolinska Institutet, Stockholm, Sweden; Department of Pediatric Surgery, Karolinska Institutet, Stockholm, Sweden; Department of Pediatric Surgery, New Children’s Hospital, Helsinki, Finland; Department of Pediatric Surgery, University of Helsinki, Helsinki, Finland; Specialist Neonatal and Paediatric Surgery, Great Ormond Street Hospital, London, UK; Institute of Child Health, University College London, London, UK; Specialist Neonatal and Paediatric Surgery, Great Ormond Street Hospital, London, UK; Department of Paediatric Surgery, Institute of Child Health, London, UK; Institute of Child Health, University College London, London, UK; Department of Paediatric Surgery, Institute of Child Health, London, UK

**Keywords:** HSCR, bowel function, urological function, sexual function, fertility, quality of life

## Abstract

**Background:**

There has been an increasing number of single-centre studies describing the long-term outcomes of patients with Hirschsprung disease. This study aimed to systematically review the literature on long-term bowel, urological, and sexual functional outcomes, fertility and quality of life in adults with Hirschsprung disease.

**Methods:**

A PROSPERO-registered systematic review of the English literature was conducted for studies published up to July 2025 that reported functional outcomes beyond childhood (≥16 years) for patients who had undergone surgery for Hirschsprung disease. Centres were contacted individually for secondary analyses of patient-level data on bowel function score, Gastrointestinal Quality of Life Index, and Short Form 36 questionnaire. Data were analysed and compared with those from healthy controls in the studies retrieved and from a reference healthy population. Hirschsprung disease clinical and surgical variables were correlated with these outcomes of interest in a patient-level analysis.

**Results:**

Fifty-three manuscripts fulfilled the inclusion criteria of 4277 papers retrieved. Patients with Hirschsprung disease had a greater likelihood of constipation (odds ratio 9.27, 95% confidence interval (c.i.) 4.78 to 18.06) and soiling (odds ratio 2.76, 1.96 to 3.89) compared with healthy controls. They scored lower on the Gastrointestinal Quality of Life Index (mean difference −5.21, 95% c.i. −9.53 to −0.89; *P* = 0.020). There were no significant differences in Short Form 36 domain scores except for physical functioning (mean difference −6.30, −8.74 to −3.87; *P* < 0.001). At a patient level, longer-segment disease (*P* < 0.001) and redo pull-through surgery (*P* = 0.002) were associated with a poorer bowel function score. Short form 36 scores were lower in women across six of eight domains; similarly, Gastrointestinal Quality of Life Index scores were lower in women (*P* < 0.001) and in patients with longer-segment disease (*P* < 0.001).

**Conclusion:**

Among patients with Hirschsprung disease, women, those with longer-segment disease, and patients who underwent redo surgery may be at risk of poorer quality of life.

## Teaser

This systematic review assessed long-term outcomes in patients with Hirschsprung disease (HSCR), focusing on bowel, urological, and sexual function, fertility, and quality of life. Patients with HSCR were more likely to experience constipation, soiling, and lower quality-of-life scores (especially women and those with severe disease), with physical role limitations and poorer Gastrointestinal Quality of Life Index scores being prominent. These findings emphasize the need to address the functional impairments and challenges faced by patients, particularly women and those with more severe forms of the disease, during their transition to adult care.

## Introduction

Hirschsprung’s disease (HSCR) is a congenital disorder characterized by intestinal aganglionosis, extending proximally from the internal anal sphincter^1^. Patients usually present in the neonatal period with intestinal obstruction, or occasionally later with chronic constipation. Definitive diagnosis requires histopathological evaluation of a rectal biopsy^2^. Management is surgical and involves resection of the affected bowel with a pull-through of the ganglionated proximal bowel to the anus^3^.

Outcomes for patients with HSCR have improved as surgical techniques have advanced^4,5^. In the short term, the length of hospital stay is reduced, with fewer postoperative complications^4,5^. However, long-term bowel dysfunction persists, and difficulties with fertility, and urological and sexual function have been reported in adult patients^6–9^. These functional impairments adversely affect the psychosocial well-being of children and adults^10–12^, and have lifelong implications that are relevant to healthcare providers.

Data on the aforementioned issues in adults with HSCR are scant in the literature. Furthermore, most studies featured patients from one centre only and so were limited to small sample sizes, which lack external validity.

The present study had two main objectives. The first was to conduct a systematic review of the literature to pool large amounts of data quantitatively and qualitatively, and quantify the impact of surgery for HSCR on adults. The second aim was to collate patient-level data and undertake secondary analyses, looking more closely at bowel function score and quality-of-life (QoL) scores to correlate functional and psychological aspects experienced by adults with HSCR.

## Methods

This systematic review adhered to the PRISMA 2020 checklist^13^. It was preregistered in the International Prospective Register of Systematic Reviews (PROSPERO) (CRD42019131530), and published before data analysis.

### Search strategy

A search was performed in PubMed (MEDLINE), the Cochrane Library, Web of Science, and clinical trials registries on 2 July 2020. The following search terms were run (‘Hirschsprung’s’ OR ‘HSCR’) AND (‘Outcomes’ OR ‘Quality of Life’ OR ‘Bowel Function’ OR ‘GIQLI’ OR ‘SF-36’). An updated search was completed on July 2025. Reference lists were also searched.

### Eligibility criteria

The literature search was restricted to papers in the English language; however, foreign language papers were translated where possible for the extraction of quantitative data. There was no restriction by publication date because of the rarity of the disease.

Patients comprised those who had undergone surgery for HSCR and with reported outcomes beyond childhood (defined as ≥ 16 years). In studies with mixed populations, only data on adult patients with HSCR were used. For the urological meta-analysis only, a pragmatic limit of ≥ 15 years was applied to obtain sufficient data points. Studies documenting results for patients with intellectual impairment were analysed separately.

In accordance with the PROSPERO registration, studies with less than 30% prospective follow-up or small sample sizes (< 20 patients overall or < 5 adults) were excluded. However, these papers were still included in the qualitative review but not in the meta-analysis.

The search was carried out by two independent reviewers and the results merged; duplicate citations were discarded. Titles and abstracts were screened by two reviewers, and conflicts were resolved by mutual agreement. The full text of the remaining articles was retrieved and data extracted using a standardized form. The same protocol was followed for the updated search, which identified three additional papers.

### Data extraction and outcomes of interest

General data on study characteristics (country, study design, and date of publication) and participant characteristics (diagnoses, method of recruitment, age range, total number of participants and adult participants) were extracted. Primary outcomes of interest were bowel function (constipation, faecal accidents and soiling, stoma use or antegrade colonic enemas (ACEs)) and quality of life (QoL). Secondary outcomes were urological function, sexual function, and fertility. For primary and secondary outcomes, both qualitative and quantitative data were extracted. Data extraction was not limited to a specific tool of measurement. Not all papers had to measure all outcomes to be included. If studies had overlapping patient cohorts, data on each outcome were extracted only once.

For the patient-level regression, corresponding authors were contacted for anonymized data sets of information on patient-level demographics (age, sex) and disease characteristics (HSCR segment length (short, long, total colonic aganglionosis (TCA)), pull-through type, presence of stoma, need for redo surgery); and outcomes in terms of bowel function, assessed using the bowel function score (BFS)^5,9,14–16^ and QoL, measured using Short Form 36 (SF-36^®^) and the Gastrointestinal Quality of Life Index (GIQLI)^7,9,14,15,17–19^.

### Risk-of-bias assessment

All included studies were reviewed for intrastudy and interstudy heterogeneity. Study quality was measured by three independent assessors using the National Heart, Lung and Blood Institute Quality Assessment tool for Cross-Sectional and Observational Cohort studies^20^. Each component question was colour-coded depending on whether it had been demonstrated or not. Studies with two or fewer missing components were given a rating of good, those with three missing were given a rating of intermediate, and those with four or more missing were rated poor. Heterogeneity was analysed mathematically using Cochrane’s *Q* and *I*^2^. Studies were included in the meta-analysis unless there was evidence of serious methodological flaws from the quality assessment.

### Statistical analysis

Review Manager (RevMan, Version 5, The Cochrane Collaboration) was used for the odds ratio (OR) meta-analysis for bowel function (constipation, soiling) and QoL (GIQLI and individual components of SF-36^®^). RStudio was used for proportional meta-analyses of bowel function (stoma, ACE) and urological function (urinary incontinence). The restricted maximum likelihood random-effects model with logit transformation was used to minimize bias^21–24^. Control data were collected primarily from included studies. If control data were not extractable, published data from the healthy population of Finland was used for bowel function^25^, and country-specific controls from the general population for SF-36^®26–29^. Owing to the large degree of heterogeneity in the reporting methods for sexual function and fertility, these findings were described qualitatively but no further statistical analyses were undertaken.

For the patient-level analysis, univariate analyses were carried out. Overall BFS and GIQLI, GIQLI subdomains, and individual BFS question items were analysed by segment length, patient sex, pull-through type, and need for redo pull-through surgery. To standardize for sex and country, SF-36^®^ data underwent Z-transformation to sex-matched control data for each country^26–29^. Data were divided by sex for each subdomain, and z-scores for men and women were compared, using the Kruskal–Wallis test with Dunn’s correction, to delineate domains in which women may be at additional risk of adverse outcomes.

Multivariable linear regression for these factors (segment length, patient sex, pull-through type, need for redo pull-through surgery), as well as patient age, was performed to identify independent correlates with overall BFS score. Because the presence of a stoma did not preclude patients from taking the GIQLI, this was also included in the multivariable analysis for these outcomes. As SF-36^®^ has eight subdomains and is not designed to have these pooled, multivariable analyses were not undertaken for these data.

To identify a correlation between functional outcomes (as defined by the BFS) and QoL outcomes (as defined by GIQLI and SF-36^®^), analyses were performed both by linear regression (generating an *R*^2^ value) as well as by categorizing outcomes into good (BFS ≥ 17), intermediate (BFS 13–16) and poor (BFS ≤ 12 or living with stoma/ACE), and using Kruskal–Wallis with Dunn’s test for multiple comparisons.

## Results

Of the 4277 papers retrieved, 53 eligible publications were included in this review (*Fig. S1*). The majority of these were cross-sectional studies, implemented at various paediatric surgical centres across multiple countries. The age of included patients with HSCR ranged from 15 to 77 years (*Table 1*). Controls were used from the individual studies if available. These included both healthy controls with no other pathologies, or controls with other pathologies but no anorectal disease. Assessment of study quality showed that 74.5% had a poor bias rating, 21.5% had intermediate bias rating, and 4% had good bias rating.

**Table 1:** Study characteristics

Reference	Year	Country	Study design	Population	Type of surgery	Adults as proportion of total cohort	Age(years)	Control
Amerstorfer *et al*.^30^	2015	Austria	Cross-sectional	TCA	Sauer’s method	3 of 8	17, 19, 33	No
Athanasakos *et al.*^31^	2004	Australia	Cross-sectional	HSCR	Soave 29Duhamel 38Unknown 5	6 of 72	30 patients < 319 patients 7–1217 patients 13–186 patients > 18	No
Aworanti *et al.*^32^	2016	Ireland	Cohort	HSCR	Transanal Soave with split in posterior wall of rectum	2 of 51	Unknown	Yes
Baillie *et al.*^33^	1999	England	Cross-sectional	HSCR	Duhamel 80	14 of 80	> 14	Yes
Barrena *et al.*^34^	2008	BoliviaSpain	Case series	TCA	Modified Lester Martin 18Swenson 8Soave 7	18 of 33 (all modified Martin)	15–33	No
Bjørnland *et al.*^35^	2017	Norway	Cross-sectional	HSCR	ERPT 200	Not given	Not given	No
Byström *et al.*^6^	2023	Sweden	Cohort	HSCR	Unknown	597	29.6(10)*	Yes
Catto-Smith *et al*.^36^	2007	Australia	Cross-sectional	HSCR	Patients aged ≥ 12 yearsSoave 24Duhamel 3Swenson 4Unknown 1	32 aged ≥ 12 years	≥ 12	No
Conway *et al*.^37^	2007	England	Cross-sectional	HSCR	Duhamel 49	49 of 49	19.9(3.6)*	Yes
Davidson *et al*.^38^	2021	England	Cross-sectional	HSCR	Duhamel 15	17 of 32 adults with LD; 11 of 17 had Down syndrome	27 (23.5–31.5)† for population aged > 18 years	Yes
Davidson *et al*.^9^	2021	England	Cross-sectional	HSCR	Duhamel 156Swenson 19Soave 11	139 of 186	28 (4–43)†	Yes
Davidson *et al*.^8^	2021	England	Cross-sectional	HSCR	Duhamel 116	137 of 137	18–43	Yes, same population as reference 9 but data on fertility
Davidson *et al*.^39^	2025	EnglandFinlandNorwaySweden	Cross-sectional	HSCR	Duhamel 47 or endorectal pull-through 29Unknown 14	90 of 90	31.6(7.0)*	Yes
Diseth *et al*.^40^	1997	Norway	Cross-sectional	HSCR	Duhamel 19	Unclear	16 (10–20)‡	Yes
Diseth *et al*.^41^	1998	Norway	Cohort	HSCRARM	Duhamel 13	Unclear	16 (10–20)‡	Yes
Drissi *et al*.^42^	2019	British Virgin IslandsFrance	Cross-sectional	HSCR	Duhamel 12Swenson 11Soave 2Lester Martin 2Rehbein 1Panproctocolectomy IPAA 1Coloanal 1Unknown 4	34 of 34	32(16) (17–77)§	No
Fosby *et al*.^43^	2020	Norway	Cohort	HSCR	ERPT 50	Unclear	FU1 8.1 (3.4–16.6)‡FU2 15.4 (9.9–25)‡	No
Granström *et al*.^17^	2015	Sweden	Cross-sectional	HSCR	Soave 29Duhamel 5Sphincteromyectomy 2Ileostomy 1Sigmoid colostomy 1Unknown 1	39 of 39	28 (20–43)‡	Yes
Gunnarsdóttir *et al*.^19^	2010	Sweden	Cross-sectional	HSCR	Duhamel 38Martin for LS 3Ileorectal for LS 1	42 of 42	28.5 (18–45)‡	Yes
Gustafson *et al*.^7^	2019	Sweden	Cohort	HSCR	Unknown 69	69 of 69	37.8 (35.5, 22–58.5)¶	Yes
Hartman *et al*.^18^	2004	The Netherlands	Cross-sectional	HSCRARM	Unknown 142	142 of 142	Unknown	Yes
Heikkinen *et al*.^44^	1995	Finland	Cross-sectional	HSCR	Duhamel 71Swenson 20Sate-Rehbein 5Soave 4Unknown 2	102 of 102	31.4(6.9)*	Yes
Heikkinen *et al.*^45^	1997	Finland	Cross-sectional	HSCR	Swenson 7State-Rehbein 4Soave 4Duhamel 39	Examined 54 of 54Eligible 132Responded to questionnaire 102Manometry45	Examined 29(7.2)*Responded 31.4(6.9)*	Yes
Hoel *et al.*^46^	2021	Norway	Cross-sectional	HSCR	Duhamel 16Unknown 1	17 of 17	29 (19–43)‡	No
Hoel *et al.*^47^	2023	Norway	Cross-sectional	HSCR	ERPT 35	Unknown	14.9 (12–18.3)‡	No
Ieiri *et al*.^48^	2010	Japan	Cohort	HSCR	Duhamel 43	43 of 43	33 (19–55)‡	No
Jarvi *et al*.^14^	2010	Finland	Cross-sectional	HSCR	Duhamel 69Rehbein 10Swenson 6Soave 2Coloanal 2	89 of 89	43 (36–51)†	Yes
Judd-Glossy *et al*.^49^	2022	Germany	Cross-sectional	HSCRARM	Unknown	16 of 16 with HSCR	32.3(10)*	No
Livaditis^50^	1981	Sweden	Case series	HSCR	Duhamel 28	Unknown of 28	15–28	No
Meinds *et al*.^51^	2019	The Netherlands	Cross-sectional	HSCR	Duhamel 210Rehbein 73Unknown 63	173 of 346	17–45	Yes
Neuvonen *et al*.^52^	2015	Finland	Case series	HSCR	ERPT 146	Unknown	15 (3–33)‡	No
Neuvonen *et al*.^53^	2017	Finland	Cohort	HSCR	ERPT 23Unknown 36	34 of 59	Overall 15 (9–21)†LUTS 14 (9–21)†Sexual function 22 (18–24)†	Yes
Neuvonen *et al*.^15^	2017	Finland	Cross-sectional	HSCR	TEPT 15TEPT with laparotomy or laparoscopy 55Ileoanal PT 3Definitive endostomy 3Unknown 3	18 of 79	15 (4–32)‡	Yes
Niramis *et al*.^54^	2008	Thailand	Cohort	HSCR	Swenson 7Duhamel 25Unknown 146	36 of 178	(> 15–20 years after operation) 16.2–29	No
Onishi *et al*.^55^	2017	Japan	Cohort	HSCR	Transabdominal Soave 16	16 of 16	25 (19–37)‡	No
Reding *et al*.^56^	1997	Belgium	Case–control	HSCR	Swenson with colostomy 16Swenson–Pellerin without colostomy 27Duhamel 1Martin 3Swenson–Delovers 2Swenson–Boley 2Ileostomy only 2	Unknown	1.2–21.5	Yes
Romero *et al*.^57^	2011	Germany	Cross-sectional	HSCR	ERPT 24Soave 10Swenson 3Rehbein 15Duhamel 1	49 of 99	1–35	No
Sherman *et al*.^58^	1989	IrelandFranceUSA	Cross-sectional	HSCR	Unknown	Unknown	Unknown	No
Sood *et al*.^59^	2018	Australia	Cohort	HSCR	Soave 46Swenson/Duhamel/Unknown 4	3 of 58	18–18.72	Yes
Stam *et al*.^60^	2006	The Netherlands	Cross-sectional	Mixed population of chronic disease	Unknown 72	72 of 72	24.1 (20–30.8)#	Yes
Stenström *et al*.^16^	2017	Sweden	Cross-sectional	TCA	Swenson 6Soave 5Short soave 3J pouch 6Duhamel 3Rehbein 2Stoma 2	3 of 27	9.5 (0.7–20)‡	No
Suita *et al*.^61^	1998	Japan	Cross-sectional	HSCR	Z-shaped anastomosis 99	49 of 99	1–35	No
Söderström *et al*.^62^	2024	SwedenDenmarkNorwayFinland	Cross-sectional	HSCR	Endorectal pull-through 54Swenson 6Duhamel 39Rehbein 21Ileoanal anastomosis with J pouch 1Posterior sagittal anorectoplasty 1Other/unknown 47	169 of 169	32 (25–41)**	No
Thakkar *et al*.^63^	2017	England	Cross-sectional	HSCR	Duhamel 70Soave 2	1 of 72	Unknown	No
Tran *et al*.^64^	2018	Belgium	Cross-sectional	HSCR	Soave 40MIS(T)ERPT 13	28 of 52	16.2 (5–28)#	Yes
Trinidad *et al*.^65^	2023	USA	Cross-sectional	HSCRARM	Unknown 19	19 of 19	Men > 18	No
van den Hondel *et al*.^66^	2015	The Netherlands	Cohort	HSCRARM	Rehbein 41Duhamel 1Unknown 12	36 of 54	26 (22–33)†	Yes
Verkuijil *et al*.^67^	2022	The Netherlands	Cross-sectional	HSCR	Duhamel 207Soave 1Rehbein 69Swenson 1Transanal pull-through 52	Unknown of 330	Familial 19 (8–42)‡Non-familial 17 (8–45)‡	No
Wildhaber *et al*.^68^	2005	USA	Cross-sectional	TCA	Unknown	Unknown	Mean FU 17.5 years but no ages	No
Witvliet *et al*.^69^	2017	The Netherlands	Cohort	HSCRARM	Unknown 10	10 of 10	27.9 (17–64)#	Yes
Witvliet *et al*.^70^	2018	The Netherlands	Cohort	HSCRARM	Duhamel 29Rehbein 7Martin 1	37 of 37	Men 27.3††Women 27.1††25–33	Yes
Xiong *et al*.^71^	2015	China	Cross-sectional	HSCR	Heart-shaped anastomosis 92No operation 2Stoma only 3Sphincterotomy/myomectomy 2Swenson 10Soave 57	92 of 92	Soiling 25.9††Non-soiling 27.7††	Yes
Yanchar *et al*.^72^	1999	Canada	Cohort	HSCR	No operation 2Stoma only 3Sphincterotomy/myomectomy 2Swenson 10Soave 57Duhamel 31	11 of 107	11 patients > 15 Oldest patient not stated at FU	No

Values are *mean(standard deviation, s.d.), †median (interquartile range, i.q.r.), ‡median (range), §mean(s.d.) (range), ¶mean (median, range), #mean (range), **mean (i.q.r.), ††mean. TCA, total colonic aganglionosis; HSCR, Hirschsprung’s disease; ERPT, endorectal pull-through; LD, learning disability; IPAA, ileal pouch anal anastomosis; FU, follow-up; LS, long segment; ARM, anorectal malformation; LUTS, lower urinary tract symptoms; TEPT, transanal endorectal pull-through; PT, pull-through; MIS(T)ERPT, minimally invasive (transanal) endorectal pull-through.

### Bowel function

#### Constipation

Studies^9,31,35–37,44,46,48,61,63,64^ showed that patients with HSCR had higher rates of constipation than healthy controls^7,14,17^; however, the incidence and severity of constipation were lower in groups with older patients. Patients with an associated syndrome or learning disability were more likely to encounter constipation symptoms, regardless of the type of pull-through they had undergone^31,38,52^. Patients with familial HSCR were more than twice as likely to experience constipation^67^. Meta-analysis conducted on 300 patients showed that those with HSCR were more likely to have constipation requiring oral or rectal medication, with an OR of 9.27 (95% confidence interval (c.i.) 4.78 to 18.06) compared with controls (*Fig. S2a*).

#### Faecal accidents and soiling

A wide range of faecal accidents and soiling definitions was used. These were grouped together for qualitative analysis, which showed that, overall, rates were higher for patients with HSCR than controls^7,14,33,51^. Patients with syndromic associations or associated learning disability were again more likely to encounter soiling and incontinence symptoms^9,31,35,43,58,64^. Across all patients, faecal accidents and soiling affected social life^48,59,71^, ability to attend school^36^, choice of occupation^35^, and sexual function^65^. This associated impact on QoL reinforced the need to support continence management actively in patients with HSCR^32,68^. In terms of anal manometry, two studies^40,45^ used anal manometry as a measure of bowel function, and found that both anal canal resting pressure and the increment during squeeze were lower in 73 patients with HSCR than in 44 controls. Using Rintala’s BFS, 247 patients with HSCR had a greater likelihood of reporting soiling than healthy controls, with an OR of 2.76 (95% c.i. 1.96 to 3.89) (*Fig. S2b*).

#### Stoma and ACEs

A small percentage of adult patients with HSCR had stomas (3.67 (95% c.i. 2.38 to 5.19)%; *I*^2^ = 0%, *P* = 0.450), and 1.58% (0.02 to 4.67)% used an ACE (*I*^2^ = 31%, *P* = 0.230) (*Fig. S2c*,*d*).

#### Patient-level analysis

**Fig. 1:**
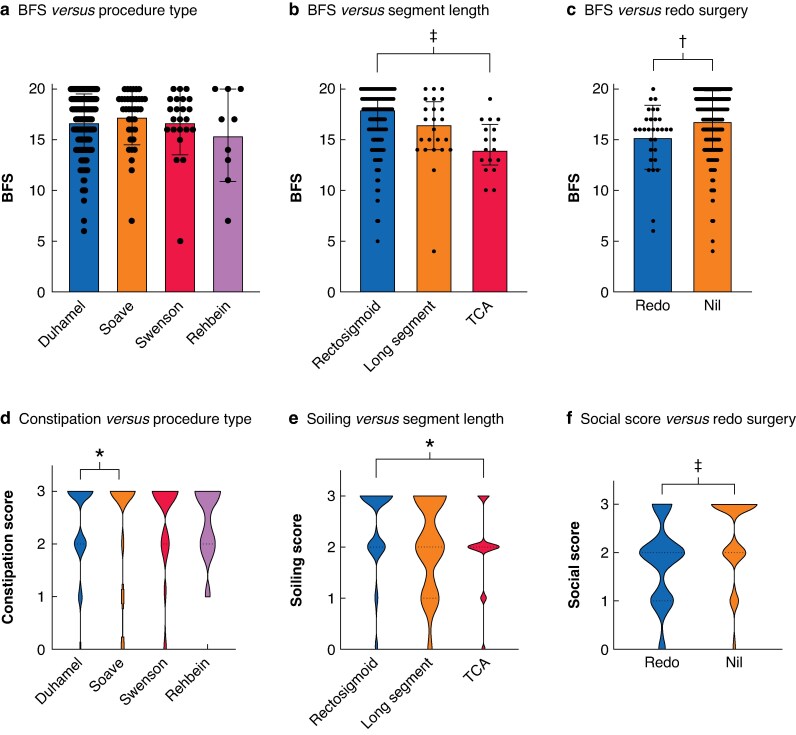
Patient-level analysis for bowel functional score (BFS) **a** Type of procedure and BFS; **b** Length of disease segment and BFS; **c** Redo surgery and BFS; **d** Type of operation and constipation rates; **e** Disease segment and soiling; **f** Redo surgery and social score. *P < 0.05; **P < 0.01; ***P < 0.001; ****P < 0.0001.

Of ten studies that used the BFS, data from seven, across three centres (UK, Sweden, and Finland) were collated for patient-level analysis, amounting to 284 patients. Some 51.1% of the cohort had normal bowel function (BFS ≥ 17), but 11.3% had a poor outcome with BFS ≤ 12 or a permanent stoma or ACE. The type of pull-through procedure did not affect the overall BFS (*Fig. 1a*). Poorer scores were noted in patients with total colonic disease (*Fig. 1b*) and those who had undergone redo surgery (*Fig. 1c*).

Analysis of individual components of the BFS showed that the Duhamel operation was associated with higher rates of constipation (*Fig. 1d*). Long-segment disease was associated with an increased prevalence of soiling (*Fig. 1e*). Bowel function-related social difficulties, according to the BFS subdomain, were associated with redo surgery (*Fig. 1f*).

In multivariable linear regression, older age at time of survey (*P* = 0.023), female sex (*P* = 0.019), longer segment length (*P* < 0.001), and redo surgery (*P* = 0.002) were all associated with a worse functional outcome (*Table S1*).

### Urological function

Urological function was described in 12 studies, using varying definitions^6,7,17,39,42,44,48,50,55,58,62,66^. The most common definition was urological incontinence (11 studies), followed by lower urinary tract symptoms (6). Data could be extracted for meta-analysis of urinary incontinence from six studies (539 patients). Some 4.37 (95% c.i. 0. to 15.97)% of adult patients with HSCR experienced some level of urinary incontinence (*I*^2^ = 96%, *P* < 0.01) (*Fig. S3* and *Table S2*).

### Sexual function

Sexual function was reported in 12 studies (*Table S3*). A range of definitions was used, including impaired erection (9 studies)^7,8,47,53,58,62,65,66,70^, impaired ejaculation (4)^7,8,66,70^, inability to climax sexually (3)^8,39,66^, dyspareunia (3)^8,39,66^, and sexual function/satisfaction (5)^39,53,62,66,70^.

The rate of erectile dysfunction ranged from 0 to 26%^58,62^ and of ejaculation difficulties from 0 to 3%^8,66^; difficulties reaching climax were reported in 9% of male patients^8^; dyspareunia was reported in half of women^8^; and sexual function was impaired in 40–53% of women with HSCR^39,66,70^.

### Fertility

Fertility was reported in nine studies (*Table S4*)^6,8,39,42,48,53,55,61,62^). Most of these used the rate of having children as a marker of fertility^6,42,48,55,61,62^, with some only reporting children with married parents^48,55,61^. The rate of children among fathers with HSCR was comparable to that in the normal population^6,62^, but women with HSCR had fewer children per individual and were older at the birth of the first child compared with the normal population^6^. A spontaneous conception rate of 83% was shown in male patients in two studies^8,62^, which reported that all 63 successfully conceived, of whom 9.5% sought medical assistance to conceive. In an all-female cohort, 44% sought medical assistance to conceive and 49% of those attempting to conceive had involuntary childlessness^39^.

### QoL

QoL was reported in 22 studies^7,9,14,15,17–19,30,38,42,44,46,51,60,64–67,69–72^, but only 7^7,9,14,15,17–19^ used SF-36^®^ scores (in 406 patients) or GIQLI scores (in 325 patients). HSCR was reported to have a negative effect on both physical and psychosocial well-being, with higher levels of school absenteeism, unhappiness or anxiety, food restrictions, peer rejection, and later psychosexual development^46,60,64^. However, other studies^42,51,69,71^ described higher educational level and favourable QoL outcomes for patients with HSCR. Bowel function influenced QoL^51,64,66,71,72^, but timing and type of operation did not^5,14^. Familial HSCR was associated with better QoL despite worse bowel function^67^, and syndromic disease had little to no impact on QoL despite generally worse functional outcomes^38^.

A meta-analysis showed lower GIQLI scores for patients with HSCR *versus* controls with a mean difference (MD) of −5.21 (95% c.i. −9.53 to −0.89; *P* = 0.020) (*Fig. S4a*). There were no significant differences in SF-36^®^ subscores except for physical functioning (MD −6.30, −8.74 to −3.87; *P* < 0.001) (*Fig. S4b*).

**Fig. 2:**
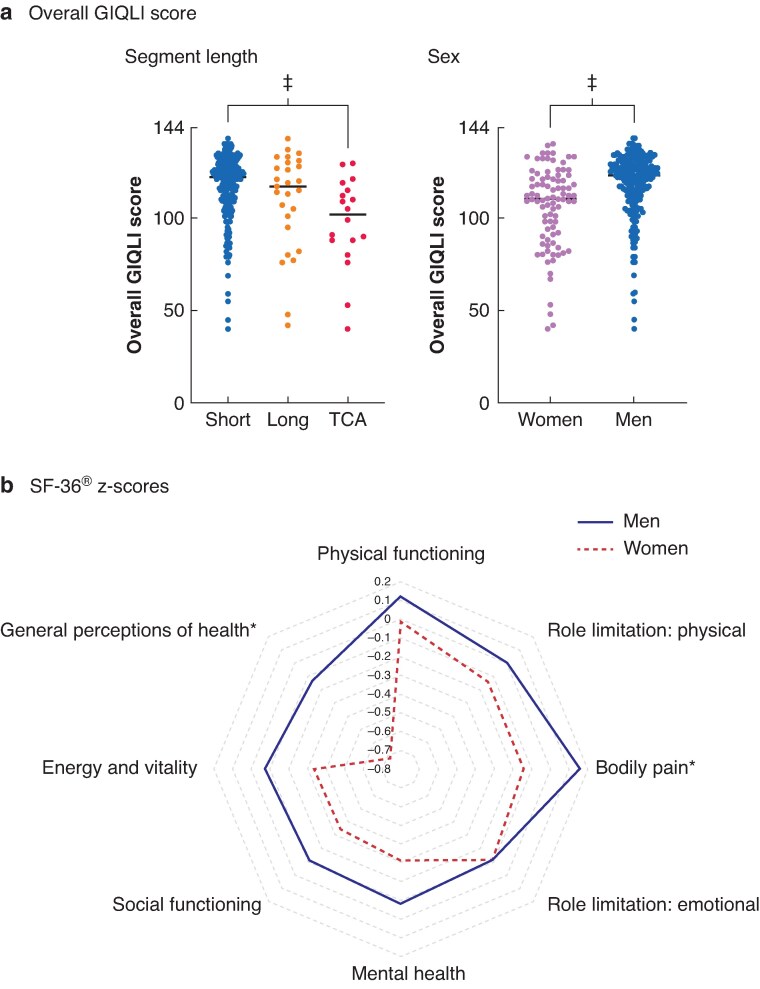
Patient-level analysis for quality of life **a** Overall Gastrointestinal Quality of Life Index (GIQLI) score^9,14,15,17^ in relation to diseased segment length and patient sex; values are shown for individual patients with a bar at the associated median value. **b** Radar plot z-scores for Short Form 36 (SF-36^®^) domains^7,9,15,17,18^. *P < 0.050, ‡*P* < 0.001 (Mann–Witney test or Kruskal–Wallis test with multiple comparison (Dunn's)).

Patient-level data sets on GIQLI and SF-36^®^ were obtained from four centres (327 patients) and five centres (447) respectively. GIQLI scores were lower in women (MD 11, 7 to 15; *P* < 0.001) and in those with longer-segment disease (Kruskal–Wallis 14.1, *P* < 0.001) (*Fig. 2a*). These remained significant on multivariable analysis of factors associated with lower GIQLI subdomain scores (*P* < 0.001 for both). SF-36^®^ scores were lower in women across six of eight domains. Women had negative z-scores across all domains compared with men, who had positive z-scores in the domains physical functioning, bodily pain, and role limitation: physical. The z-score differed significantly between men and women for general perception of health (z-score −0.72 for women *versus* −0.13 for men) and bodily pain (−0.14 *versus* 0.16 respectively) (*Fig. 2b*).

### Correlation between functional score and QoL

**Fig. 3:**
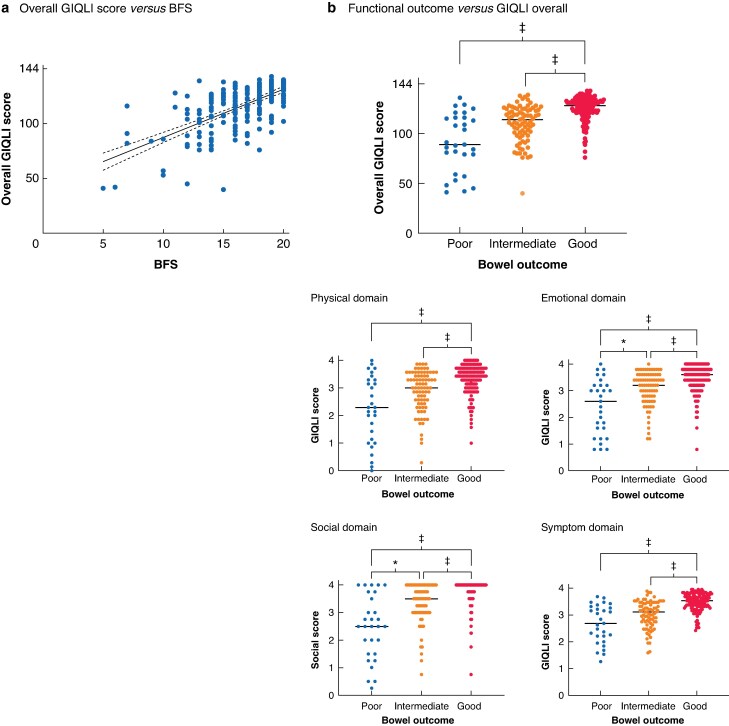
Analysis of functional scores and quality of life **a** Overall Gastrointestinal Quality of Life Index (GIQLI) score in relation to bowel function score (BFS); dots, solid lines, and dashed lines indicate mean and 95% confidence interval of simple linear regression. **b** Functional outcomes *versus* GIQLI scores. *P < 0.050, ‡*P* < 0.001 (Kruskal–Wallis test with multiple comparisons (Dunn's)).

There was a clear correlation between GIQLI scores (overall and subdomains) and BFS (*Fig. 3a*), with positive correlation coefficients (*R*^2^ 0.479–0.675; *P* < 0.001 for all). With correction for multiple testing, differences between poor (BFS < 12 or living with stoma/ACE) and intermediate (BFS 13–16) bowel outcomes were found only in emotional and social subdomains, with no difference in overall, symptom, and physical scores (*Fig. 3b*).

**Fig. 4:**
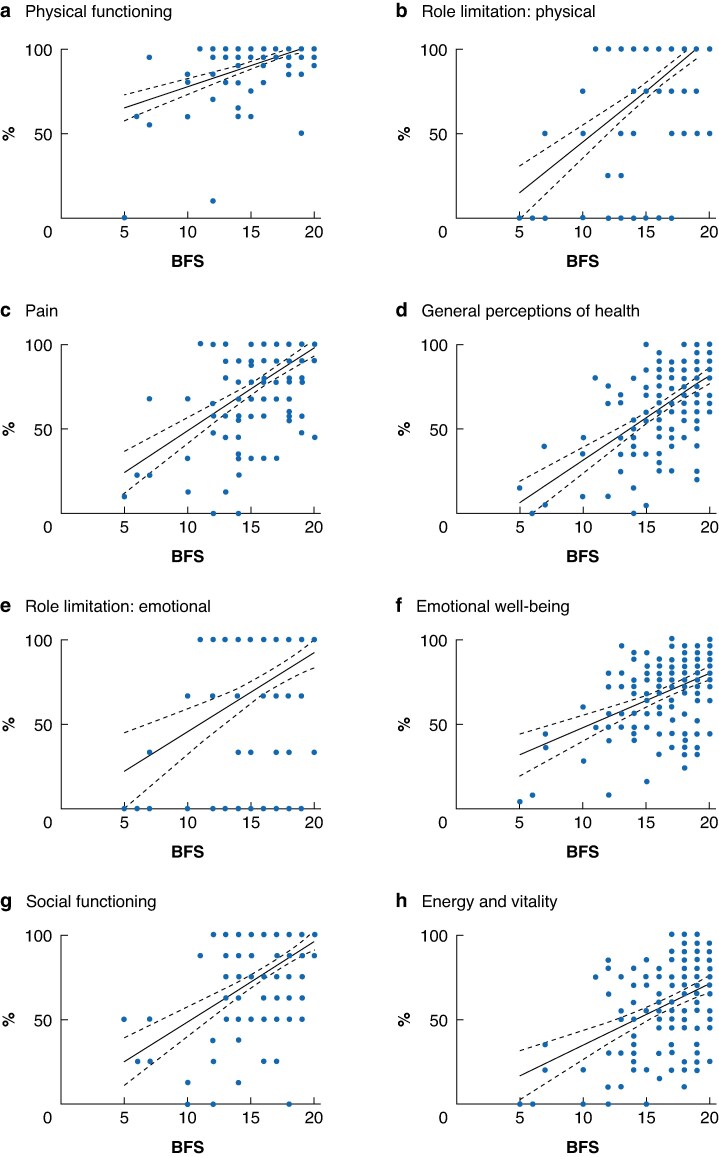
Analysis of BFS and SF-36^®^ Bowal function scores (BFS) for subdomains of Short Form 36 (SF-36^®^): **a** physical functioning, **b** role limitation: physical, **c** pain, **d** general perceptions of health, **e** role limitation: emotional, **f** emotional well-being, **g** social functioning, and **h** energy and vitality. Dots represent individual data points, solid lines dashed lines indicate simple linear regression mean and 95% confidence interval.

Subdomain scores for the SF-36^®^ were compared with BFS in the same way. Similar to findings for the GIQLI, there was a statistically significant correlation across all subdomains (Spearman’s ρ 0.297 to 0.572; *P* < 0.001 for all) (*Fig. 4*).

## Discussion

This study found that adult patients with HSCR had higher rates of constipation, soiling, and stomas than healthy controls, but most had adequate an overall BFS (≥ 17). QoL in adults with HSCR is strongly influenced by bowel continence, initial length of segment, and female sex. There is a paucity of research in urological and sexual function and fertility, with many studies limited to men only.

As expected, bowel function was worse for patients with HSCR than for controls^7,14,17,33,51^, but improved with age^9,31,35,36,41,44,46,48,51,54–56,61,64^. This age-dependent improvement could be explained by changes in the disease itself or improved management of symptoms by the patient^9,51^. The Duhamel operation was associated with higher rates of constipation, in concordance with previous studies^73^, although overall functional scores did not differ. Functional impairment was more prevalent in longer-segment disease and in those requiring redo surgery, emphasizing the importance of a successful index procedure. Lastly, reported function was worse in those who were older at time of survey, or women - which is a finding that has been noted in studies of functional outcomes in other gastrointestinal conditions^74,75^.

Urological function was generally favourable in patients with HSCR, although a wide range of definitions was used. It was seldom the primary outcome of the included studies, and sometimes only collected as a baseline parameter, which makes the reported results less reliable. In addition, men and women were often analysed together^7,17,42,44,48,50,53,55,58^, which risks presenting a misleading picture as urinary tract function differs between the sexes in the general population.

Sexual function and fertility were poorly investigated, particularly in women. Sexual function was assessed by means of non-comparable scores, or scores with no scientific bearing (such as marital status), with few exceptions. Erection and ejaculation difficulties were comparable to those in general population; however, adult patients with HSCR had high rates of dyspareunia (50%), sexual distress (37.5%), and sexual functional issues (53%). This underscores the need for further research and the importance of highlighting this issue in the transition to adult care^8,66,70,76–81^.

Gastrointestinal-oriented tools for evaluating QoL showed that HSCR had a negative impact on QoL, whereas general instruments showed no correlation between the two at a population level. This suggests that adults with HSCR develop coping mechanisms for their symptoms, as noted previously^80^. Factors that affect both gastrointestinal-related and generic QoL include bowel function, female sex, and length of diseased segment (longer associated with worse gastrointestinal-related QoL outcomes). QoL among women also diverged more from the general population than that of their male counterparts, suggesting a greater lifelong impact of HSCR in women.

Despite advancements in surgery and medicine, patients with HSCR have worse outcomes than healthy controls. However, the studies used a wide range of definitions, which makes this comparison challenging. To address this, there should be international standards for evaluating long-term outcomes, and the continued development and use of international HSCR registers such as the European register.

Although symptoms improve with age, a significant proportion of adults with HSCR are still adversely affected throughout their lives. To address these negative long-term effects, these patients would benefit from a smooth transition of care to adult specialists. Transition occurs at a vulnerable time for patients, and can result in worse health outcomes^82,83^. Furthermore, parents are often no longer as available to support the young person and assist their decision-making. Future studies should consider transition requirements and how these are met across different healthcare systems.

The main limitation of this review is the large degree of heterogeneity between studies. Some studies^11,15,30–34,38,46,49,55,59,63,65,70,72^ also had a very small subset of their population within the adult range, creating a risk of bias owing to the small sample size. Adults were also sometimes defined using different ages, so not all data could be included in meta-analysis. Additionally, in several studies, men and women were analysed together, despite the well known differences between their reported outcomes.

Overall, this study effectively evaluated functional outcomes in a large number of patients, generating robust and generalizable results. The patient-level meta-regression also enabled detailed analyses and subgroup analyses, including comparisons between sexes.

Overall, living with HSCR as an adult requires a balance between the physiological demands of the condition and the psychological resilience needed to maintain a positive QoL^84^. Research plays a pivotal role in refining treatment approaches, highlighting the need for ongoing medical care and shedding light on psychosocial aspects. As more individuals with HSCR navigate the complexities of adulthood, the integration of research findings into their care will be instrumental in fostering a holistic and informed approach to managing this lifelong condition.

## Supplementary Material

zraf107_Supplementary_Data

## Data Availability

Study-level qualitative and quantitative synthesis was performed on publicly available data from published literature. Anonymized patient-level data would be made available upon reasonable request, via corresponding author, to the individual centre(s) responsible for those specific data.
