# Supplementary material for: Long-term effects of Hirschsprung disease in adults: meta-analysis and patient-level regression study
Source: BJS Open. 2025 Nov 4;9(6):zraf107. doi: 10.1093/bjsopen/zraf107 (PMC12586849; doi:10.1093/bjsopen/zraf107)
Supplement: zraf107_Supplementary_Data [file zraf107_supplementary_data.docx]

**Long-term Effects of Hirschsprung Disease in Adults: A Systematic Review, Meta-Analysis and Patient-Level Regression Study**

Marta de Andres Crespo^1*^, Cornelia Byström^2*^, Athanasios Tyraskis^3^, Annika Mutanen^4,5^, Pernilla Stenström^6^, Esther Hartman^7^, Johan Danielsson^8^, Simon Eaton^1,9^, Paolo De Coppi^1,3,9^, Anna Löf Granström^2^, Tomas Wester^2^, Mikko Pakarinen^4,5^, Joe Curry^3^, Stavros Loukogeorgakis^1,3,9^, Joe Davidson^1,9^.

*Joint first authors

1. University College London, London, UK.
2. Karolinska Institutet, Stockholm, Sweden.
3. Great Ormond Street Hospital, London, UK.
4. New Children’s Hospital, Helsinki, Finland.
5. University of Helsinki, Helsinki, Finland.
6. Lund University, Lund, Sweden.
7. Tilburg University, The Netherlands.
8. Institution of Women’s and Children’s Health, Uppsala University, Sweden.
9. Institute of Child Health, London, UK.

**Corresponding Author**:

Joseph Davidson

Department of Paediatric Surgery, Zayed Centre for Research, GOS-UCL Institute of Child Health, London, UK

[joseph.davidson@ucl.ac.uk](mailto:Joseph.davidson@doctors.org.uk)

**Supplementary Materials - Index**

| **Supplementary Figures and Tables** |  |
| --- | --- |
| **Figure S1.** PRISMA Diagram. | *Pag. 3* |
| **Figure S2.** Meta-Analysis for Bowel Function. **S2(a)** Constipation, **S2(b)** Soiling, **S2(c)** Stoma and **S2(d)** ACE | *Pag. 3* |
| **Figure S3.** Meta-Analysis for Urologic Function | *Pag. 4* |
| **Figure S4.** Meta-analysis for QoL. **S4(a)** GIQLI; **S4(b)** SF-36: Role limitation: Physical; **S4(c)** SF-36: Role limitation: Emotional; **S4(d)** SF-36: Bodily pain; **S4(e)** SF-36: Mental health; **S4(f)** SF-36: Social functioning; **S4(g)** SF-36: Energy and vitality; **S4(h)** SF-36: General Perception of Health. | *Pag. 5-6* |
| **Table S1.** Multiple Linear Regression for the outcome of BFS (Score 1-20) | *Pag. 7* |
| **Table S2.** Urologic function Summary | *Pag. 8* |
| **Table S3.** Sexual Function Summary | *Pag. 9-10* |
| **Table S4.** Fertility summary. | *Pag. 11* |

**Supplementary Figures and Tables**

**Figure S1.** PRISMA Diagram.

**
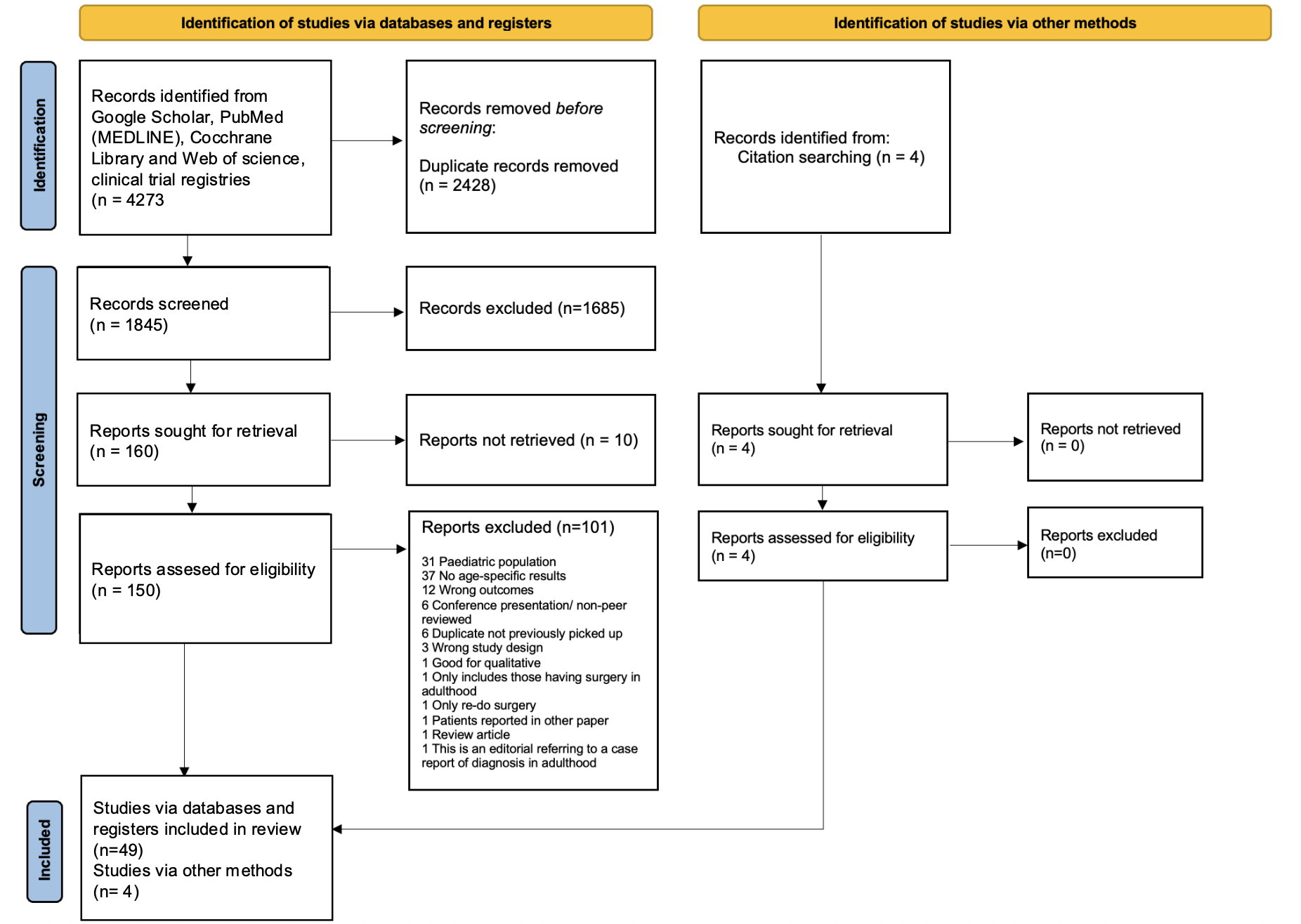
**

**Figure S2.** Meta-Analysis for Bowel Function.

**S2(a) S2(b)**


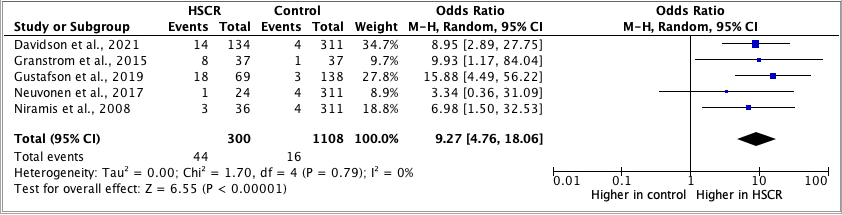

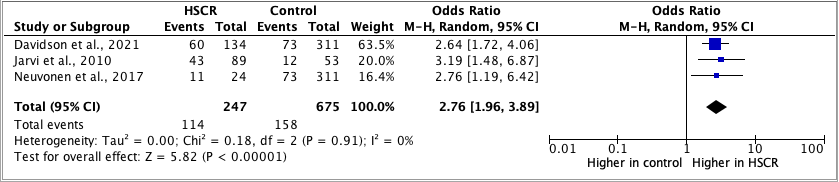


**S2(c) S2(d)**


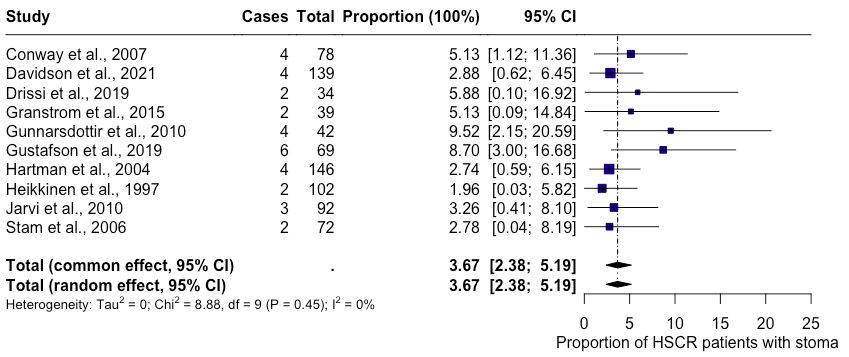

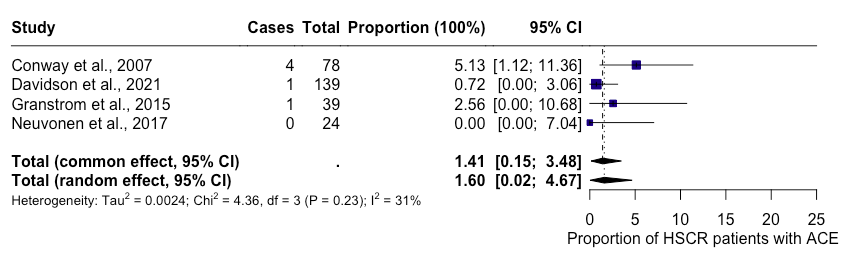


**S2(a)** Constipation, **S2(b)** Soiling, **S2(c)** Stoma and **S2(d)** ACE.

**Figure S3.** Meta-analysis for urologic function; urinary incontinence.


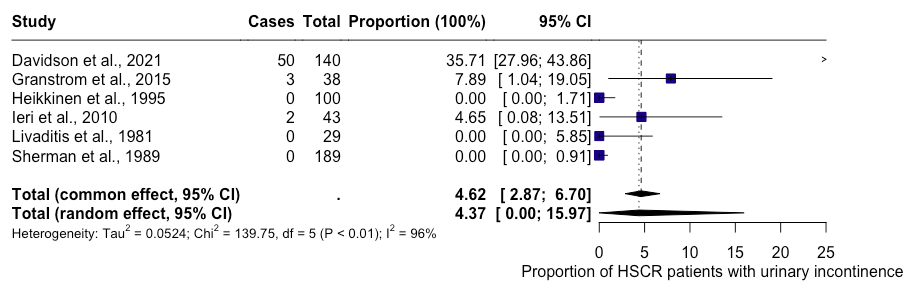


**Figure S4.** Meta-analysis for QoL.

**S4(a)**
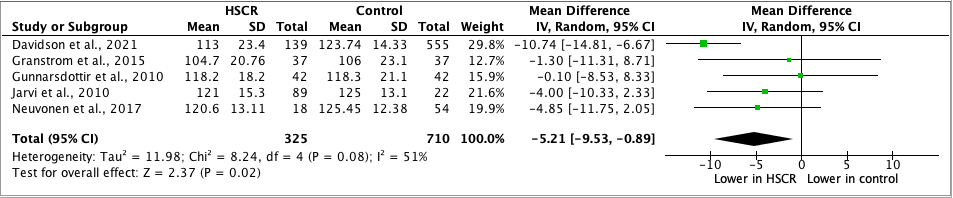


**S4(b)**
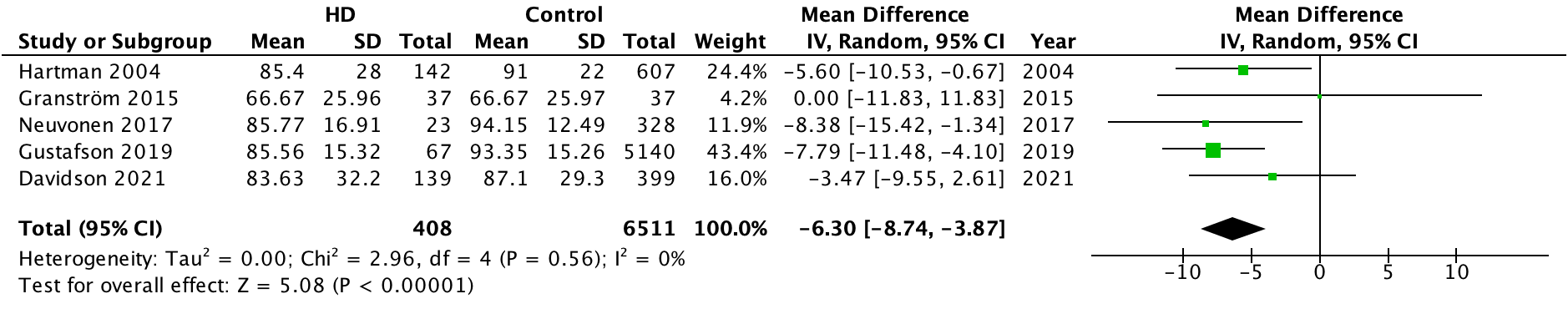


**S4(c)
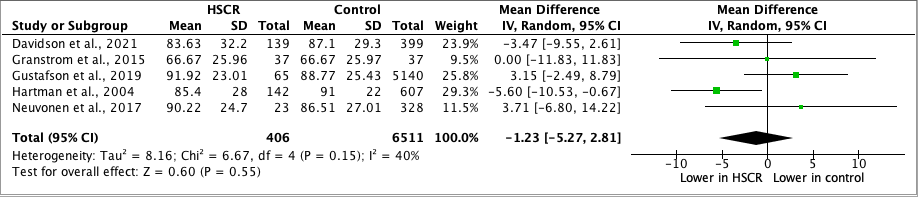
**

**S4(d)
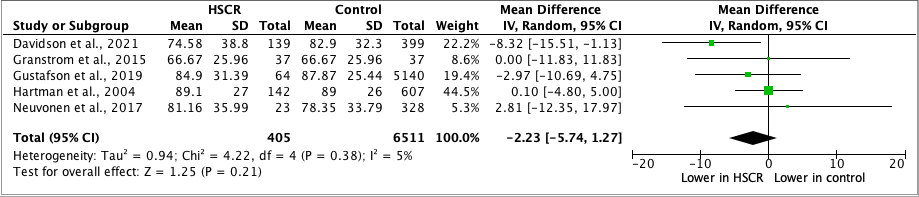
**

**S4(e)
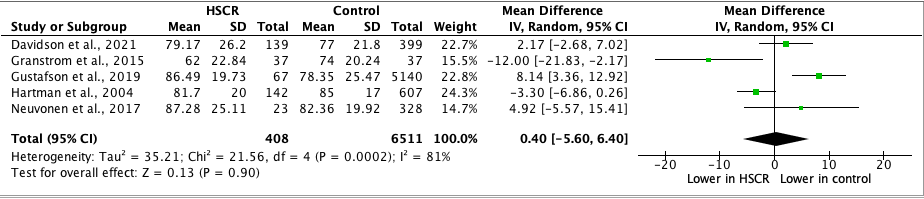
**

**S4(f)
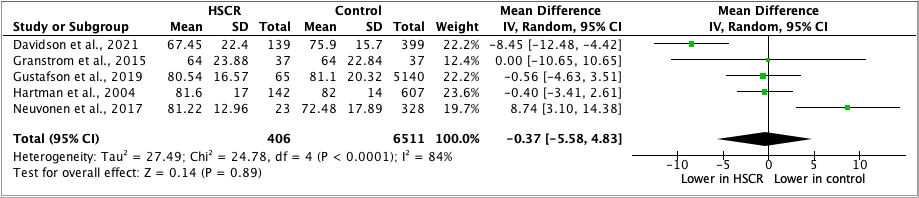
**

**S4(g)
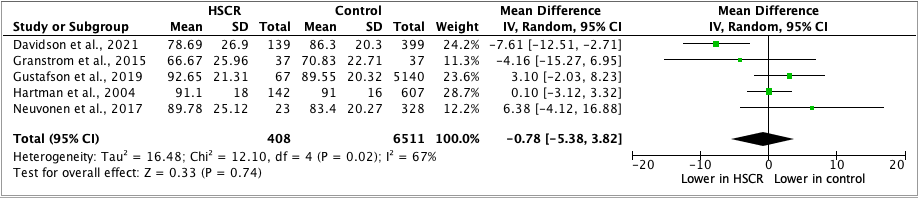
**

**S4(h)
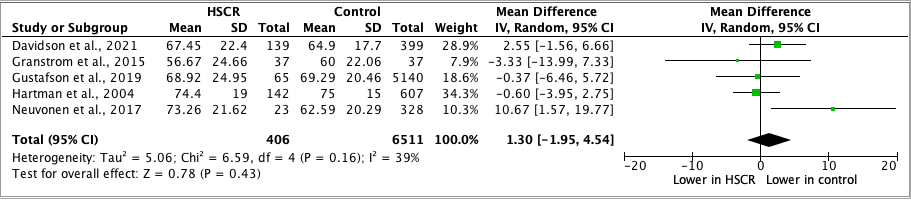
**

**S4(i)
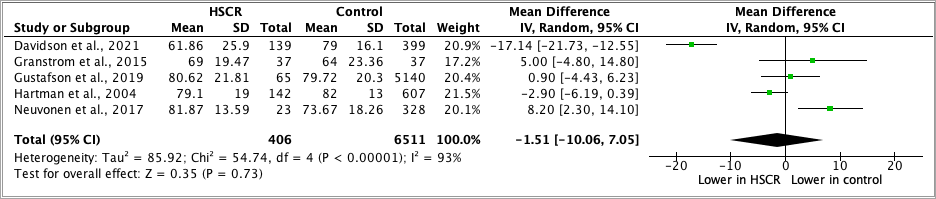
**

**Figure S4.** Meta-analysis for QoL. **S4(a)** GIQLI; **S4(b)** SF-36 Physical Functioning **S4(c)** SF-36: Role limitation: Physical; **S4(d)** SF-36: Role limitation: Emotional; **S4(e)** SF-36: Bodily pain; **S4(f)** SF-36: Mental health; **S4(g)** SF-36: Social functioning; **S4(h)** SF-36: Energy and vitality; **S4(i)** SF-36: General Perception of Health

**Table S1.** Multiple Linear Regression for the outcome of BFS (Score 1-20)

|  | Unstandardized | | Standardized |  |  |
| --- | --- | --- | --- | --- | --- |
|  | B | Std. Error | Beta | t | p-value |
| Regression constant | 18.83 | 8.11 | - | 23.221 | <0.001 |
| Age at survey (per year) | -0.038 | 0.017 | -0.142 | -2.295 | 0.023 |
| Female Sex | -0.931 | 0.393 | -0.143 | -2.368 | 0.019 |
| Segment (Standard, Long, TCA) | -1.033 | 0.310 | -0.204 | -3.332 | <0.001 |
| Type of Pull-Through | -0.017 | 0.224 | -0.005 | -0.077 | 0.939 |
| Redo Surgery | -1.556 | 0.496 | -0.191 | -3.136 | 0.002 |

**Table S2.** Urologic function summary

| **Study** | **Evaluation tool** | **Incontinence** | **Other Urinary tract symptoms** | **Comments** |
| --- | --- | --- | --- | --- |
| **Byström et al. (6)** | ICD-code for urinary incontinence registered in the Swedish national patient register | Overall: 36/597 (6%) |  |  |
| **Davidson et al. (39)** | Modified Danish PSS | At least Daily incontinence: 2/140 At least Weekly incontinence: 14/140 At least Occasional incontinence: 50/140 **Male** At least Daily incontinence: 0/99 At least Weekly incontinence: 4/99 At least Occasional incontinence: 22/99 **Female** At least Daily incontinence: 2/41 At least Weekly incontinence: 10/41 At least Occasional incontinence: 28/41 | Any LUTS: 96/140 UTI last year: 14/140 **Male:** Any LUTS: 61/99 UTI last year: 4/99 **Female:** Any LUTS: 35/41 UTI last year: 10/41 | Data collected from original author. Patients ≥ 18y.o. |
| **Davidson et al. (39)** | Modified Danish PSS | 9/16 |  | Patients with learning disabilities |
| **Drissi et al. (60)** | HAQL: Urinary continence.  Domain score: 0-100 | “Urinary continence” was only marginally affected |  | No rates or scores available, only graph |
| **Granström et al. (16)** | Questionnaire. Question on Incontinence and difficulty emptying bladder | Overall: 3/38 | Difficulty emptying bladder: 3/38 |  |
| **Gustafson et al. (7)** | Questions with answers on a four-graded scale (1=None, 2=Some, 3=Quite a lot, 4=Very much)  1) Problems with urinary voiding  2) Problems with urinary leakage | No difference in problems with urinary leakage.  HSCR: 1.2 (1, 1–4), control: 1.2 (1, 1–3), median (range), p=0.6241. | Higher incidence of problems with urinary voiding in the HSCR-group.  HSCR: 1.3 (1, 1–3), control 1.16 (1, 1–3), median (range), p=0.0230 | Rates of incontinence/other urinary tract symptoms not available |
| **Heikkinen et al. (32)** | Questionnaire.  Question on urinary symptoms and urinary continence | Overall: 0/100 | - | Questions on urinary symptoms not defined. |
| **Ieiri et al. (34)** | Interview + questionnaire | Overall: 2/43 | Frequent urination: 3/43 Micturition pain: 2/43 | Questions on urinary function not defined. |
| **Livaditis et al. (59)** | Interview, questionnaire, patient records | Overall: 0/29 | - | Questions on urinary function not defined. Patients ≥15 y.o. |
| **Onishi et al. (45)** | Questionnaire. Question on Frequent urination and Micturition pain. | - | Frequent urination: 0/16 Micturition pain: 0/16 |  |
| **Sherman et al. (49)** | Interview | Overall: 0/184 |  | Questions on urinary function not defined. |
| **Söderström et al (62)** | IPSS | - | Urinary tract symptoms:  mild (132, 80.5%), moderate (28, 17.1%),  severe (four, 2.4%) |  |
| **Van den Hondel et al. (61)** | HAQL: Urinary continence.  Domain score: 0-100 | Median (range): Males: 100 (16.7) Females: 100 (8.3) |  | Rates of incontinence/other urinary tract symptoms not available |

**Table S3.** Sexual Function Summary

| **Study** | **Participants and evaluation tool** | **Erectile dysfunction** | **Ejaculatory dysfunction** | **Other measurement sexual function** | **Comments** |
| --- | --- | --- | --- | --- | --- |
| **Davidson et al. (8)** | HSCR (n): 132  Female: 38  Male: 94  EHS SQOL | 3/94 (3%) | Issues with sexual climax: 8/94 (9%) | Frequent dyspareunia in 19 of 38 sexually active women (50%).  Anorgasmia: 1/94 Retrograde ejaculation: 2/94  SQOL median score, male: 100 (86.4–100). 19% and 14% lower than 1s.d. and 2s.d. of normal population.  SQOL median score, female: 77 (62.5–85), 47% and 22% scoring below 1s.d. and 2S.D. respectively (<1s.d. versus men: OR 3.85, 95% C.I.:. 1.65–9.04, P=0.002; <2S.D. | Compared with normative summary data no evidence for reduced SQOL in the men, whereas the women reported significantly lower SQOL scores.  Overlap of female patients between Davidson et al. (8) and Davidson et al. (66). Only data on female patients from Davidson (66) have been used. |
| **Davidson et al. (66)** | HSCR (n): 90  Female:90  FSFI | - | - | Lower (worse) scores in female HSCR patients in the domain “pain” compared to healthy controls.  40% of HSCR patients scored <26/36 (impaired sexual function) on FSFI compared 27% of controls (non-significant difference). | Overlap of female patients from Davidson et al. (8) and Davidson et al. (66). Only data on female patients from Davidson (66) have been used. |
| **Gustafson et al. (7)** | HSCR (n): 71  Female: 13  Male: 58  Questionnaire. | No significant differences regarding erectile problems. (median score, no rate reported) | Significantly more problems with ejaculation. (median score, no rate reported) | No statistical differences in the bowel function’s impact on either interest for or being able to take pleasure in sexual activities | Questions on: - Bowel function has a negative impact on interest of sex - Bowel function limits taking pleasure in sexual activity - Problems with erection (males only) - Problems with ejaculation (males only) |
| **Hoel et al. (64)** | HSCR (n): 15  Female: -  Male: 15  Semi-structured interview on sexual function and LUTS, questionnaire on LUTS (DAN-PSS). | 0/15 (0%) | 1/15 (6.7%) |  | Patients aged ≥15 years |
| **Hoel et al. (33)** | HSCR (n): 17  Female: 8  Male: 9  Qualitative interviews |  |  | Men expressed good sexual self-esteem and few problems with intimate relationships and fertility. Women more reluctant to discuss intimacy, had more problems with sexual relationships and were concerned about telling partners about HD. The females who had experienced problems to conceive, wondered if the infertility was related to HD. |  |
| **Neuvonen et al. (62)** | HSCR (n): 24  Female: 8  Male: 16  EHS GIQLI Sexual health questionnaire | 0/16 (0%) |  | Sexual satisfaction, female: 71% vs 83% controls Sexual satisfaction, male: 79% vs 67% controls |  |
| **Sherman et al. (49)** | HSCR (n): 194  Female: -  Male: 194  Interview | 0/194 (0%) |  |  | “194 of the older males asked if they had difficulty with erection or ejaculation and all answered in the negative" - age not specified but assumed to be ≥16y based on age description. |
| **Stam et al. (67)** | HSCR (n): 72  Female: 12  Male: 60  The Course of Life Questionnaire |  |  | HD patients had later psychosexual development compared to reference population. |  |
| **Söderström et al.**  **(62)** | HSCR (n): 169  Male: 169  SQoL-M  IIEF-5 | 40/152 (26%) | - | Median IIEF-5 score: 24 (21-25).  Median SQoL-M: 95 (69-100).  Lower IIEF-5 and SQoL-M in adolescent patients, patients with poor bowel function and poor urinary tract function. |  |
| **van den Hondel et al. (61)** | HSCR (n): 33  Female: 15  Male: 18  HAQL IIEF FSFI FSDS | 2/20 (11%) |  | IIEF not affected compared to reference population. Lower FSFI (p=0.03; with lower satisfaction and pain scores) - 53% report sexual function issues - 6 (40%) below 2 SD, 3 report distress (20%) | Mean age for reference population was 55y. |
| **Witvliet et al. (63)** | HSCR (n): 37  Female: 9  Male: 28  IIEF FSFI FSDS | 1/28 (4.2%) |  | Sexual function/satisfaction: FSFI: 50% HSCR women scored 26 or lower FSDS: 37.5% scored 15 or higher |  |
| **Trinidad et al. (53)** | HSCR (n): 19  Female: -  Male: 19  IIEF MSHQ | 2/12 (16.7%) |  | IIEF: Median score 25 (22.5-25). MSHQ: median score 15 (IQR 14-15), 0/18 bothered score. MSHQ-EjD survey overall: 15 (IQR 14-15) | Both patients with erectile dysfunction: mild-moderate |

**Table S4.** Fertility summary.

| **Study** | **Evaluation tool** | **Fertility** | **Comments** |
| --- | --- | --- | --- |
| **Byström et al. (6)** | Prevalence of having children | Total: 191/597 (32%) Male: 149/454 (32.8%) Female: 42/143 (29.4%) - sign lower than reference population in all groups. | Crossreference between patients with HSCR in Swedish national patient register and Swedish medical birth register |
| **Davidson et al. (8)** | Successful attempts to concieve | 38/53 (successful attempts to conceive)  3 additionally men and 3 adittionally women had children through IVF  Males 17% unable to spontaneously conceive with 8% successful with IVF. Females 53% unable to conceive spontaneously of which 18% were successful with IVF. | Clearly different rates between male and female in spontaneous conception.   SQOL scores lower in women.  Compared with normative summary data no evidence for reduced SQOL in the men compared to control, whereas the women reported significantly lower SQOL scores: 71.9(20.7) versus 90.7(15.0) respectively; P < 0.001, Hedge’s g 1⁄4 1.0 (large effect).  Overlap of female patients from Davidson et al. (8) and Davidson et al. (66). Only data on female patients from Davidson (66) have been used. |
| **Davidson et al. (66)** | Successfull attempts to concieve | 45/90 female HSCR patients had tried to conceive.  Unable to conceive within 1 year of trying: 21/45  Unable to conceive within 2 years of trying: 12/45  Accessed fertility services: 20/45  Successful IVF-attempts: 11/17  Involuntary childlessness: 22/45 | All-female cohort.  Overlap of female patients from Davidson et al. (8) and Davidson et al. (66). Only data on female patients from Davidson (66) have been used. |
| **Drissi et al. (60)** | Prevalence of having children | 12/34 | No difference from control group |
| **Ieiri et al. (34)** | Prevalence of having children | 13/19 | Prevalence of children only investigated in married patients. (50% patients of married patients in their 20s had children, 62.5% of the married patients in their 30shad children, and 100% of the married patients in their 40s) |
| **Onishi et al. (45)** | Prevalence of having children | 3/5 | Prevalence of children only investigated in married patients (5/16 married) |
| **Neuvonen et al. (62)** | Successful attempts to conceive (males only) | 3/3 (successful attempts to conceive, only males) |  |
| **Suita et al. (36)** | Prevalence of having children | 8/12 | Prevalence of children only investigated in married patients (12/30 married) |
| **Söderström et al. (62)** | Successful attemps to concieve, prevalence of having children, medical assisted conception. (Males only). | 63/169 (37.3%) had children.  8/63 reported failure to concieve within one year.  5/63 reported failure to concieve within two years.  6/63 needed medical assistance to concieve. | Comparable results to the general population. |
